# Dictionary of Medical Science

**Published:** 1894-01

**Authors:** 


### Book Notices.

A DICTIONARY OF MEDICAL SCIENCE.—Containing a full explanation of the various subjects and terms of Anatomy, Physiology, Medical Chemistry, Pharmacy, Pharmacology, Therapeutics, Medicine, Hygiene, Dietetics, Pathology, Surgery, Bacteriology, Ophthalmology, Otology, Laryngology, Dermatology, Gynecology, Obstetrics, Pediatrics, Medical Jurisprudence and Dentistry, etc., etc. By Robley Dunglison, M. D., LL. D., late professor of Institutes of Medicine in the Jefferson Medical College of Philadelphia. Edited by Richard J. Dunglison, A. M., M. D. New (21st) edition, thoroughly revised, greatly enlarged and improved, with the Pronunciation, Accentuation and Derivation of the Terms. In one magnificent imperial octavo volume of 1181 pages. Cloth, $7.00; leather, $8.00. Philadelphia: Lea Brothers & Co., 1893.

For a number of years the medical profession has been demanding a revised edition of "Dunglison's Medical Dictionary," and we now have it larger, better and more complete than ever before. The page has been enlarged, about one hundred pages and forty-four thousand new words have been added; pronunciation has been introduced for the first time, derivation of words are given, the definitions have been expanded to include much valuable and practical information not always easily found elsewhere and everything obsolete has been excised. In no previous edition have the changes and additions been so great. The valuable tables for which "Dunglison" has been noted in previous editions have been retained and enlarged and improved upon. With all the changes and additions that have been made the work is still comprised in a convenient volume.

In a work of this magnitude it would be a matter of great surprise if a few errors did not occur, but we believe that this one is as free from them as any that can be found. The phonetic pronunciation is an improvement over the former editions which gave merely the accentuation, but a far better plan could have been adopted. If the author had used a system of pronunciation similar to that used in Webster's dictionaries there could be no chance of making a mistake, and the value of the book would have been greatly enhanced thereby. It is so complete in words and so rich in definitions that the matter of a system of pronunciation seems very insignificant in comparison. It is decidedly the best we have and we can say with the publishers: "It is safe to call *Dunglison's Medical Dictionary* an indispensable book for students, practitioners, pharmacists, dentists and all concerned with any of the medical sciences." H.

